# Boosting Hydrogen Evolution Kinetics with MoS_2_-Decorated TiO_2_ Nanotubes

**DOI:** 10.1021/acsomega.5c06367

**Published:** 2026-03-25

**Authors:** Leonardo J. L. Maciel, Denilson V. Freitas, Felipe L. N. Sousa, Luana B. C. Oliveira, Otávio A. L. Alves, Francisco de A.S. Ribeiro, Giovanna Machado

**Affiliations:** † Centro de Tecnologias Estratégicas do Nordeste (CETENE), 50740-540 Recife, PE, Brazil; ‡ Universidade Federal de Pernambuco (UFPE), 50670-901 Recife, PE, Brazil

## Abstract

The development of efficient and durable nonplatinum-based electrocatalysts for the hydrogen evolution reaction (HER) remains a key challenge. This study explores the heterojunction between titanium dioxide nanotubes (TiO_2_ NTs) and molybdenum disulfide quantum dots (MoS_2_ QDs) as a viable alternative to platinum-based HER electrocatalysts. TiO_2_ NTs were synthesized via anodization, while MoS_2_ QDs were electrosynthesized chronopotentiometrically, followed by heterojunction formation via immersion/adsorption. The optimized TiO_2_ NTs/MoS_2_ QDs electrocatalyst exhibited significantly enhanced HER activity, achieving an overpotential of 617 mV at 100 mA cm^−2^ (η^100^), a notable improvement over 927 mV observed for bare TiO_2_ NTs. Electrochemical impedance spectroscopy (EIS) revealed a dramatic reduction in charge transfer resistance from 475 to 9.9 Ω after MoS_2_ QDs deposition. A Tafel slope of 106 mV dec^−1^ indicated a Volmer−Heyrovsky HER mechanism with second-order kinetics, as confirmed by kinetic modeling. Structural and morphological characterization (XRD, SEM, TEM, and EDX) confirmed the successful heterojunction formation. This work highlights the potential of TiO_2_ NTs/MoS_2_ QDs as scalable and efficient electrocatalysts for sustainable hydrogen production, offering a promising alternative to platinum-based systems.

## Graphical abstract

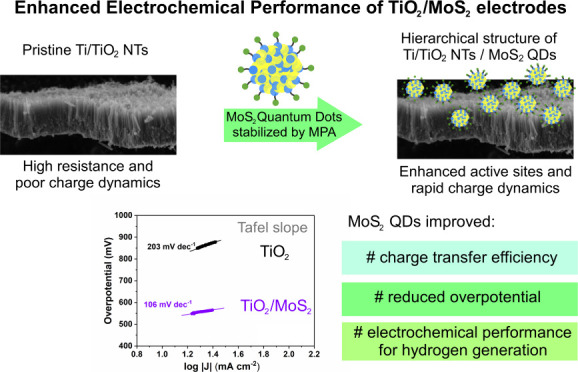

## 1 Introduction

Hydrogen gas is emerging as a key green energy carrier due to its carbon-free nature and high energy density, offering a promising alternative fuel for mitigating environmental challenges.^1^ This growing demand for clean energy has fueled interest in producing hydrogen from renewable sources, such as water, to reduce dependence on hydrocarbon-based technologies. As a result, extensive research has focused on electrocatalytic water reduction for hydrogen production.^2–4^ Although precious metals like platinum (Pt) and ruthenium are highly efficient electrocatalysts for the hydrogen evolution reaction (HER), their scarcity and high cost hinder large-scale implementation. Therefore, the search for cost-effective, durable, and high-performance HER electrocatalysts is a critical challenge for scaling up sustainable hydrogen production.^5^

In this context, transition metal oxides have gained attention as promising HER candidates due to their low cost, high surface area, corrosion resistance, catalytic activity, and long-term stability under high potentials.^1,6^ However, their practical application still faces challenges, particularly in improving the overall electrochemical water splitting efficiency, often limited by stability issues and interface transformations. Titanium dioxide (TiO_2_) has a *E*_g_^bulk^ = 3.2 eV and is renowned for its exceptional physicochemical stability across a broad pH range. However, its direct use in electrocatalytic hydrogen generation is limited by its high overpotentials. Despite this limitation, TiO_2_ surface properties are ideal for adsorbing active nanostructures, allowing the formation of heterojunctions that can significantly enhance charge transfer efficiency.^7^ Volcano plots are a valuable tool for identifying alternative materials to replace platinum (Pt) in TiO_2_/Pt composites, which are widely employed in electrocatalysis.

A promising strategy for enhanced electrocatalysis involves coupling TiO_2_ with transition metal dichalcogenides (TMDCs), materials known for their unique electronic and catalytic properties. TMDCs have demonstrated remarkable potential across various fields.^8^ In the Mo−S phase diagram, the phases MoS_2_ and Mo_2_S_3_ presented a layered structure. Typically, MoS_2_ has a S−Mo−S sandwich-layered structure held together via weak van der Waals interactions. It is analogous to graphene, in which Mo is surrounded by the S atoms through covalent bonding. The surface sensitization of TiO_2_ by MoS_2_ can systematically control the electron−hole pair recombination by acting as trapping centers of the electron.^9,10^

Molybdenum disulfide (MoS_2_) has attracted attention due not only to its tunable band gap energy (1.3−1.9 eV), thin thickness, and large surface area but also to its abundant availability, low cost, high activity, and special chemical stability.^11^ Therefore, the TiO_2_/MoS_2_ heterojunction system formed by combining TiO_2_ and MoS_2_ with a layered structure has high carrier separation and migration efficiencies, numerous active reactive sites, and a wide light harvesting range, which can significantly enhance the photocatalytic activity.^12^ Numerous techniques, including chemical vapor deposition, hydrothermal, mechanical cleavage method, liquid-phase exfoliation, and electrochemical exfoliation, have been used to construct large-area, few-layer, and atomically thin MoS_2_ nanosheets.^13–17^ The MoS_2_ structure is susceptible to the formation of sulfide vacancies, which can enhance chemical reactivity and catalytic action.^18,19^ The introduction of vacancies into the semiconductor structure leads to an increase in wider layer spacing and charge carrier mobility.^20,21^

In this work, we report on the development of a TiO_2_ NTs/MoS_2_ QDs heterojunction to reduce the overpotential and enhance the electrocatalytic activity of electrodes for the hydrogen evolution reaction (HER). MoS_2_ quantum dots (QDs) were anchored onto TiO_2_ nanotubes (NTs) via an immersion strategy, leading to physical adsorption. The resulting TiO_2_ NTs/MoS_2_ QDs electrode exhibited enhanced electron transfer and a lower Tafel slope (106 mV dec^−1^) compared to those of bare TiO_2_ NTs (203 mV dec^−1^), indicating improved catalytic efficiency. Furthermore, the TiO_2_ NTs/MoS_2_ QDs electrodes demonstrated a stable, sustained HER activity even after 12 h of continuous operation. Kinetic modeling, experimentally validated, revealed a pseudo-second-order reaction mechanism, underscoring the critical roles of surface processes and mass transport in governing the overall rate. These findings highlight the critical roles of surface processes and mass transport in governing the overall reaction rate, reinforcing the potential of TiO_2_/MoS_2_ as an efficient and durable electrocatalyst for hydrogen generation.

## 2 Experimental Section

All chemicals were of reagent grade and used without further purification. Elemental sulfur (S^0^) powder (100 mesh, 99.9%), molybdenum (Mo^0^) plate (≥99%), graphite powder (particle size < 20 μm), 3-mercaptopropionic acid (MPA, ≥98%), and AgNO_3_ (≥99.0%) were acquired from Sigma-Aldrich. Titanium foil (98.6%), monoethylene glycol (MEG, 99%), acetone (99.5%), NH_4_F (≥98.0%), NaOH (98%), KOH (98%), and NaCl (98%) were acquired from Synth. Ultrapure water (18.2 Ω cm, Milli-Q system) was used.

### 2.1 Electrochemical TiO_2_ Nanotube Growth

TiO_2_ NTs were produced via chronoamperometric anodization process, ensuring controlled oxidation of the titanium surface.^7,22^ Prior to anodization, titanium plates (2 × 10 cm, 20 cm^2^) were sequentially cleaned using a low-power ultrasonic bath in three steps: (i) dextran (10% w/w), (ii) acetone, and (iii) ultrapure water, each for 10 min. The anodization process was conducted at a constant potential of 45 V for 110 min under a controlled temperature of 25 °C, using a DC power supply (Minipa MPL-3303M). A two-electrode parallel configuration was employed, with a titanium plate as the anode and a copper plate as the cathode, both having identical exposed surface areas (38 cm^2^, sum of the exposed front and back of each electrode). The electrodes were immersed in 200 mL of an electrolyte solution containing ethylene glycol, 0.5 wt % NH_4_F, and 10 wt % ultrapure water. Following anodic growth of TiO_2_ NTs on the Ti surface (Ti/TiO_2_ NTs), the material underwent thermal treatment at 400 °C for 3 h to ensure the formation of the anatase phase and enhance mechanical strength.

### 2.2 Electrosynthesis of MoS_2_ Quantum Dots

Molybdenum disulfide quantum dots (MoS_2_ QDs) were synthesized via paired electrosynthesis following a chronopotentiometric procedure.^23,24^ The electrochemical process was performed using a PGSTAT Autolab 302N potentiostat/galvanostat (Metrohm, The Netherlands), controlled by Nova 2.1.6 software. The reaction was conducted under a constant current of −30 mA, with a total electrical charge of −46 C. The electrochemical cavity cell used for MoS_2_ QD production (Figure S1) consisted of three compartments: (a) the anodic compartment, formed by molybdenum plate to promote the Mo^4+^ formation, (b) the central compartment containing an aqueous solution of 3-mercaptopropionic acid (MPA), serving as a stabilizing agent, and (c) the cathodic compartment designed to promote HS^−^ formation, using a graphite powder macroelectrode. The cathodic macroelectrode was constructed from a polished graphite bar fixed in Teflon, forming a cavity filled with a mixture of graphite powder (3.0 mmol) and elemental sulfur (0.1 mmol). The mixture was compacted at 3.2 kg cm^−2^ for 10 min. To prevent graphite dispersion, minimize liquid junction potential, and enhance HS^−^ migration to the central compartment, a sintered glass separator, presonicated in 1.0 mol L^−1^ NaCl, was placed over the cavity.

The central compartment was filled with 20 mL of an aqueous solution containing 0.2 mol L^−1^ NaCl and 0.065 mol L^−1^ MPA, with the pH adjusted to 7 using 1.0 mol L^−1^ NaOH. The molybdenum plate (anode) was immersed in this solution, and N_2_ purging was maintained throughout the synthesis to remove dissolved oxygen and enhance mass transfer efficiency. The MoS_2_ seeds were subjected to reflux heating (10 min) and subsequently stored at 4 °C. To remove the NaCl electrolyte, the synthesized MoS_2_ QDs were purified by dialysis for 5 h using cellulose acetate membranes. The release of Cl^−^ ions through the selective membrane was monitored by the precipitation of silver chloride (AgCl), induced by the addition of 0.5 mol L^−1^ AgNO_3._

### 2.3 Sensitization of TiO_2_ NTs with MoS_2_ QDs

To facilitate the adsorption of MoS_2_ QDs onto the TiO_2_ NTs surface, an immersion-based sensitization process was conducted. Ti/TiO_2_ plates (2 × 1 cm), were immersed in a MoS_2_ QDs solution (0.5 mg mL^−1^) for varying durations of 1, 6, 12, and 24 h at a constant temperature of 4 °C and without stirring. Following sensitization, the TiO_2_/MoS_2_ electrodes were rinsed with deionized water three times with deionized water, air-dried, and stored in the dark before characterization.

### 2.4 Electrochemical and HER Measurements

All electrochemical measurements were performed using a PGSTAT Autolab 302N instrument (Metrohm, Netherlands). A three-electrode configuration was applied, where the working electrode was based on the developed interface of TiO_2_/MoS_2_ (geometrical area of 1.0 cm^−2^), platinum wire as the counter electrode, and Ag/AgCl (3.0 mol L^−1^ KCl) as the reference electrode. A 1.0 mol L^−1^ KOH solution was used as the supporting electrolyte.

All electrical potentials reported in the study were converted to the RHE scale using eq 1:

$$E_{\mathrm{RHE}}=E_{\mathrm{Ag}/\mathrm{AgCl}}+0.197+(0.059\times \mathrm{pH})$$ 1

Linear sweep voltammetry (LSV) was performed to evaluate the catalytic activity for the hydrogen evolution reaction (HER). Measurements were conducted at a scan rate of −5.0 mV s^−1^, sweeping from 0 to −2.0 V vs Ag/AgCl, to analyze the overpotential. Chronopotentiometry was carried out for 12 h at a constant current density of 10.0 mA.cm^−2^ to assess the long-term stability of the electrodes.

Electrochemical impedance spectroscopy (EIS) measurements were conducted over a frequency range from 100 kHz to 0.1 Hz at the onset potential (−1.5 V vs Ag/AgCl (3.0 mol L^−1^ KCl)) with a signal amplitude of 10 mV. To ensure accurate data analysis, the high-frequency region real impedance (RHFR) measured at the open-circuit potential (OCP) was used to apply 100% IR correction for each electrode. Equivalent electrical circuits were simulated by using Nova 2.1.6 software (Metrohm). A platinum foil electrode was used as a benchmark material with an ECSA comparable to our TiO_2_ NT-modified electrodes. The hydrogen evolution reaction measurements were performed in an electrochemical cell, PINE model RRPG329, using an Autolab PGSTAT302N potentiostat. The reaction was monitored by applying a constant current of 10 mA over 4 h in a 1.0 mol L^−1^ KOH electrolyte. The quantification of evolved hydrogen was conducted using an Agilent GC system, model 8860, equipped with HP-MOLSIEVE and HP-PLOT Q columns (both 30 m in length) and a thermal conductivity detector (TCD) for gas analysis.

### 2.5 Characterizations

The synthesized nanomaterials were characterized by structural, electronic, and interface analysis techniques to gain deeper insights into heterojunction formation and charge transfer mechanisms. UV−vis absorption spectra were acquired in transmittance and reflectance modes using an Agilent Cary 300 UV−vis spectrometer over a wavelength range of 200−800 nm, with a resolution of 1 nm. For the reflectance mode, an integrating sphere was coupled to the spectrometer to enhance measurement accuracy. The emission spectra of MoS_2_ QDs were obtained using a FluoroMax Horiba spectrofluorometer with excitation and emission slit widths set to 3 nm. Measurements were performed at various excitation wavelengths (λ_exc_) ranging from 280 to 400 nm to analyze the optical properties of the quantum dots. Time-resolved photoluminescence (TRPL) spectra of MoS_2_ QDs were acquired at an λ_exc_ of 339 nm (NanoLED, Horiba), corresponding to the maximum emission of the sample. To ensure accurate light scattering calibration, Ludox (colloidal silica standard) was used as a reference. The interfaces and relative colloidal stability of MoS_2_ QDs stabilized by 3-mercaptopropionic acid (MPA) were analyzed by using vibrational spectroscopies and electrical charge measurement techniques. The spectra were acquired using a Bruker Vertex 70 spectrophotometer in attenuated total reflection (ATR) mode. Measurements were conducted with a resolution of 4 cm^−1^ over 16 scans, covering a spectral range of 4000−400 cm^−1^. The zeta potential (ζ) of colloidal MoS_2_ QDs was determined using a Litesizer 500 (Anton Paar) via the laser light diffusion method. All measurements were conducted in triplicate (*n* = 5), and the results were averaged to ensure accuracy. The morphology of the MoS_2_ QDs was investigated by means of transmission electron microscopy (TEM) on a Morgagni TEM (FEI) at an accelerating voltage of 100 kV, coupled with an energy-dispersive X-ray (EDX) detector for elemental composition analysis. To determine the size distribution of the nanoparticles, 500 randomly selected MoS_2_ QDs were analyzed using ImageJ (version 1.50, NIH, public domain) based on TEM images.

The crystalline structures of the TiO_2_ NTs, MoS_2_ QDs, and their heterojunctions were analyzed using X-ray diffraction (XRD). Measurements were conducted on a Rigaku SmartLab SE diffractometer with Cu Kα radiation (λ = 1.54 Å), covering a 2θ range of 10°−80° with a step size of 0.01°. For powder XRD, MoS_2_ QD samples were prepared in two steps: dialysis to remove impurities, followed by lyophilization for sample dehydration. The dried samples were macerated using an agate mortar and stored in a dry atmosphere to prevent contamination. The reference patterns were obtained from the repository Crystallography Open Database (COD). The interface between TiO_2_ NTs and MoS_2_ QDs was investigated using scanning electron microscopy (SEM) to evaluate the quantum dot deposition on the TiO_2_ surface after immersion. SEM imaging was performed using a Tescan Mira3 SEM instrument at an acceleration voltage of 30 kV under various magnifications.

X-ray photoelectron spectroscopy (XPS) and in situ XPS spectra were collected using an XPS K-Alpha spectrometer (Thermo Fisher Scientific) using an Al Kα X-ray source. The calibration peak positions were adjusted using the adventitious carbon peak (C 1s) at 284.7 eV. The Open-Source software KherveFitting, version 1.6, September 25, was used to analyze the data.

## 3 Results and Discussion

### 3.1 Structural and Optical Characterization of MoS_2_ QDs

MoS_2_ seeds were synthesized via paired electrosynthesis using the chronopotentiometry method. This process relies on the reaction between cathodically generated S^2−^ and anodically generated Mo^4+^ to form the molybdenum disulfide (MoS_2_) structure. In the graphite powder macroelectrode, the reduction of elemental sulfur (S^0^) occurs through a two-electron transfer process, converting S^0^ into sulfide ions (S^2−^) (eq 2). Constant cathodic polarization generates electrostatic repulsion, continuously expelling the S^2−^ ions from the graphite powder through the glass separator. During this process, S^2−^ ions undergo hydrolysis to form *HS*_(*aq*)_^−^ species, which are chemically stable within the pH range 7−14.^8,12^

$${S^{0}}_{\left(s\right)}+2e^{-}\to {S^{2-}}_{\left(\mathrm{aq}\right)}$$ 2

$${S^{2-}}_{\left(\mathrm{aq}\right)}+H_{2}O_{\left(l\right)}\to {{\mathrm{HS}}^{-}}_{\left(\mathrm{aq}\right)}+{{\mathrm{OH}}^{-}}_{\left(\mathrm{aq}\right)}$$ 3

The anodic reaction facilitates the formation of the metal cation precursor essential for MoS_2_ nucleation. In the three-electron anodic dissolution process, Mo^0^ is oxidized to Mo^4+^ ions (eq 3). The thiol groups of 3-mercaptopropionic acid (MPA) exhibit a strong affinity for the Mo^4+^ ions in solution due to the low polarizability of both cations and anions, forming a hard−hard acid−base pair, as described by Pearson’s principle. This interaction leads to the formation of [Mo­(MPA)_*n*_]^*x*−^ complexes,^25^ which then react with the HS_(aq)_^−^ species. The reaction is governed by the solubility product reaction (*k*_sp_ = 2.2 × 10^−56^) and results in the characteristic yellowish solution characteristic of MoS_2_ seeds (eq 5).^8^ Subsequent thermal treatment under reflux promotes the growth of MoS_2_ seeds into the MoS_2_ QDs (eq 6). In addition to controlling QD growth and stabilizing the interface, MPA plays a crucial role in anchoring MoS_2_ QDs onto metal oxides, such as TiO_2_, due to the carboxyl functional groups present in its structure.^5^

$${\mathrm{Mo}}^{0}\to {\mathrm{Mo}}_{\left(\mathrm{aq}\right)}^{4+}+4e^{-}$$ 4

$$[\mathrm{Mo}(\mathrm{MPA}{)}_{n}{]}^{x-}+2{\mathrm{HS}}_{\left(\mathrm{aq}\right)}^{-}\to {\mathrm{MoS}}_{2}-{\mathrm{MPA}}_{\left(\mathrm{seeds}\right)}$$ 5

$${\mathrm{MoS}}_{2}-{\mathrm{MPA}}_{\left(\mathrm{seeds}\right)}\Delta \to {\mathrm{MoS}}_{2}-{\mathrm{MPA}}_{\left(\mathrm{colloid}\right)}$$ 6

MoS_2_ quantum dots were characterized by powder XRD, and the experimental data are presented in Figure 1A. Broadened diffraction peaks, characteristic of nanoscale scattering, were observed. The crystal structure of molybdenum disulfide (2H-MoS_2_ COD number 9007661) was identified by indexing the diffraction peak at a 2θ value of 14.1°, corresponding to the (002) plane.^26,27^ Two diffraction peaks associated with sodium chloride (NaCl, COD number 1000041) were also verified at a 2θ value of 31.8°, corresponding to the (200) plane. The NaCl electrolyte, used in electrosynthesis, is known to intercalate into the MoS_2_ structure.^28^ The obtained crystal structure is similar to that reported in works with conventional synthesis via molten salt and CVD methods.^29–31^

Bulk MoS_2_ is an indirect bandgap semiconductor; however, the quantum effects associated with exfoliation promote the transition to the direct bandgap mechanism.^32^ Thus, the impact of particle growth on their optical properties was investigated by using UV−vis absorption spectroscopy. Figure S2a illustrates the evolution of absorption bands as a function of heating time. Initially, a hypsochromic shift (blue shift) was observed, with the absorption peak shifting from λ_abs_ = 290 nm (0 min) to λ_abs_ = 288 nm (30 min). Subsequently, a bathochromic shift (red shift) occurred, moving the peak to 310 nm, after 45 min. This shift is attributed to the progressive growth and exfoliation of the MoS_2_ QDs.

The evolution of the optical bandgap (E_g_) with heating time was determined using Tauc plot extrapolation (Figure S2b), and the E_g_ vs time is presented in Figure S2c. An initial increase in E_g_ was observed, from 2.52 eV (0 min) to 2.85 eV (10 min), after which it stabilized. After the dialysis process, which was used to reduce the NaCl concentration in the solution, a further shift in the absorption bands was observed, with peaks at λ_abs_ = 317 nm and λ_abs_ = 404 nm. This shift is attributed to the increased concentration of MoS_2_ QDs, which affects their optical absorption characteristics. MoS_2_ can undergo plasmonic coupling, allowing fundamental electrons to be promoted to excited states.^33^ Figure 1B presents the absorption spectra of MoS_2_ QDs, both as-synthesized and after dialysis. The process was developed to decrease NaCl (electrolyte) in the medium. The dialysis step was performed to decrease the concentration of NaCl electrolyte in the medium. The characteristic absorption band for the semiconductor was verified at a wavelength (λ_abs_) of 400 nm. Furthermore, the Tauc plot (inset of Figure 1B) showed an optical bandgap energy of 2.84 eV, confirming the maintenance of the optical properties throughout the purification process. The decrease in electrolyte concentration leads to an improvement in colloidal stability. MoS_2_ is an indirect bandgap semiconductor, which accounts for the observation of excitation-wavelength-dependent photoluminescence (PL) emission.^34,35^ Figure 1C shows this characteristic for the range of excitation wavelengths (360−420 nm). A characteristic feature of few-layer particles is the broadening of the emission bands, exhibiting an average full width at half-maximum (FWHM) of 150 nm. This broadening is attributed to the polydispersity in particle size and the presence of point defects within the structure, both of which contribute to the formation of intragap states that facilitate charge recombination. The time-resolved PL decay spectrum at λ_exc_ = 339 nm (Figure 1D) revealed the contribution of two distinct luminescence processes—excitonic recombination and donor−acceptor intragap transitions.^36^ The purely excitonic processes exhibited a constant decay time (τ_1_) of 0.27 ns with a relative abundance (A_1_) of 59.22%. In contrast, the intragap PL processes, associated with surface recombination, presented a constant decay time (τ_2_) of 3.86 ns and a relative abundance (A_2_) of 40.78%. This underscores the significant role of excitonic processes and the contribution of oscillators to the observed luminescence.^37^

**Figure 1:**
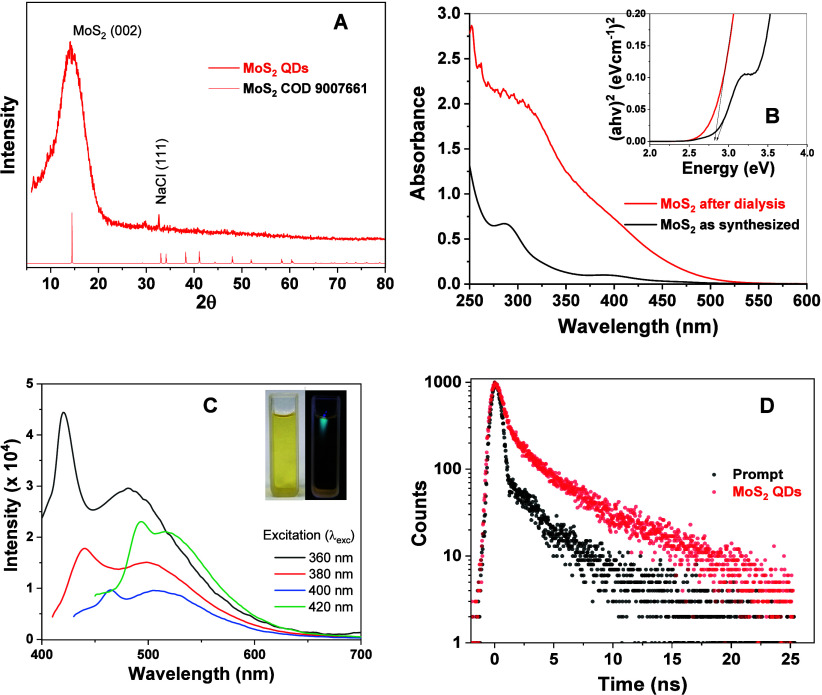
Structural and optical characterization of molybdenum disulfide QDs: (A) XRD pattern, (B) UV−vis spectra, (C) emission spectra (inset: samples under room illumination and under 405 nm excitation), and (D) time-resolved photoluminescence spectra (λ_exc_ = 339 nm, Ludox as standard).

TEM images revealed the spherical morphology of the MoS_2_ QDs, as shown in Figure 2A. The size histogram showed an average nanoparticle size of 3.2 ± 0.04 nm (Figure 2B, inset), with *n* = 500. The EDX spectrum (Figure 2B) revealed the characteristic spectral lines of S K_α1_ (2.31 keV) and Mo L_α1_ (2.29 keV), K_α1_ (17.47 keV), and K_β1_ (19.60 keV). Additionally, spectral lines corresponding to K_α1_ (0.52 keV) were observed, attributed to the stabilizers (MPA). Lines for sodium and chlorine, associated with the electrolyte, were also detected.

**Figure 2:**
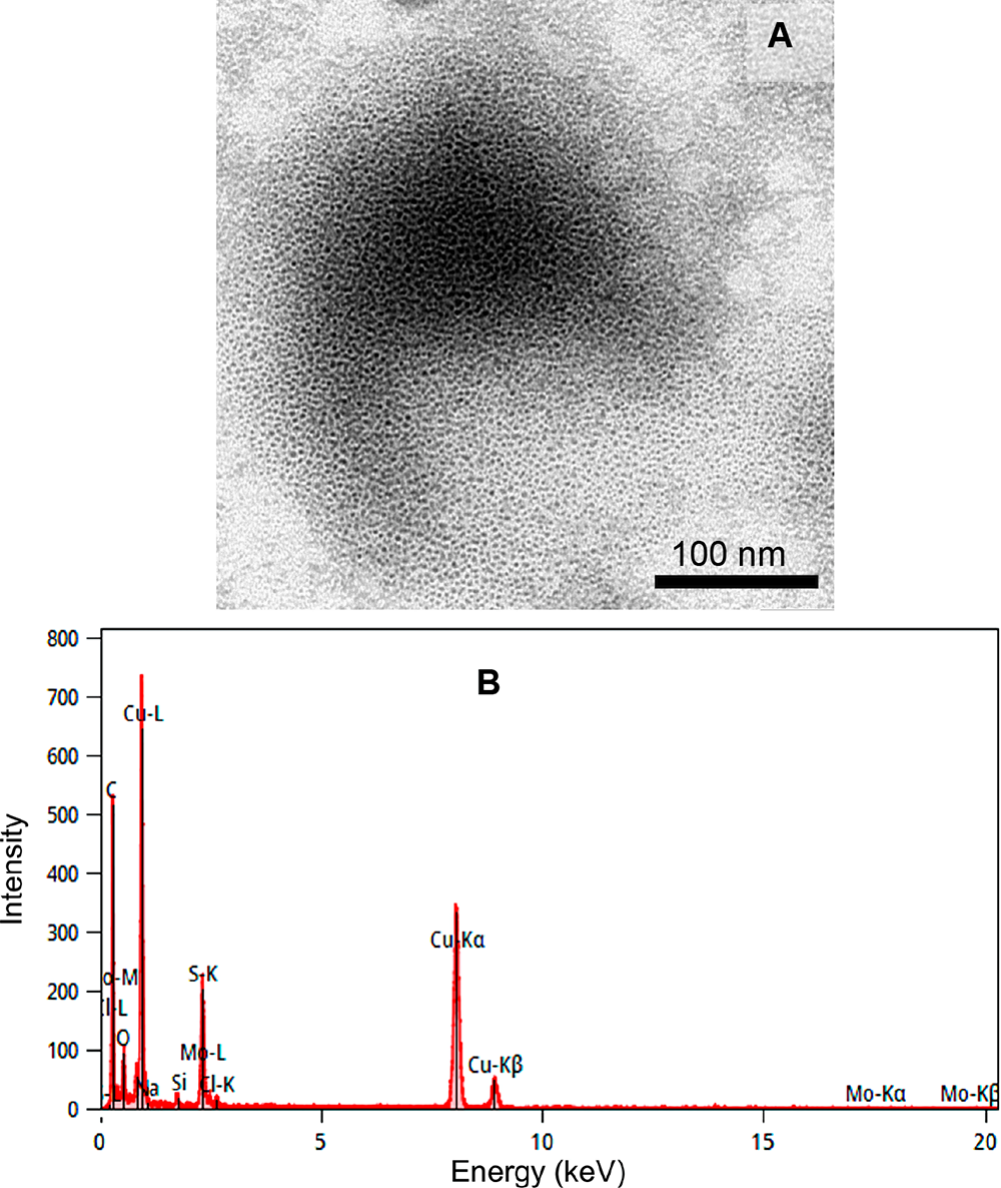
(A) TEM image obtained under potential acceleration of 100 kV and (B) energy-dispersive X-ray (EDX) spectrum of MoS_2_ QDs (inset: size histogram).

The interface between quantum dots and their surrounding solution is critical for preserving nanoscale properties and ensuring passivation against oxidative processes. MPA serves as a stabilizing agent, providing two functional groups for coordination and passivation of dangling bonds on the nanoparticle surface: thiol (−SH) and carboxyl (−COOH) groups. The preferential coordination occurs via the thiol group (−SH) due to the higher polarizability of both the cation (Mo^4+^) and anion (S^2−^), facilitating strong interactions. The FTIR spectra (Figure S5) compare pure MPA with MPA-stabilized MoS_2_ QDs, revealing distinct vibrational features. The MoS_2_-MPA QDs FTIR spectrum presented spectroscopic signals at 3366 cm^−1^, associated with O−H stretches of carboxylic groups, at 1706 cm^−1^, associated with C=O stretches, and at 1632 cm^−1^, associated with water bonding.^38^ Notably, the absence of the S−H stretching signal at 2569 cm^−1^ confirms the stabilization of MoS_2_ QDs via Mo^4+^−S­(CH_2_)_2_COO^−^ bond formation, indicating successful ligand binding between Mo­(III) centers and MPA sulfide groups.^9,39,40^

Surface potential measurements further validate the colloidal stability of the MoS_2_ QDs. Zeta potential analysis yielded a surface charge of −33.0 ± 1.1 mV, confirming the orientation of carboxylate (−COO^−^) groups on the outer surface of the nanoparticles. Furthermore, surface charge values with an absolute value greater than 30 mV indicate a highly stable colloidal system, reinforcing the effectiveness of MPA in preventing nanoparticle aggregation.^41^

### 3.2 TiO_2_/MoS_2_ Heterojunction

The heterojunction between the TiO_2_ NTs and the MoS_2_ QDs is mediated by the organic stabilizer, MPA. The preferential self-alignment of MPA can be explained by Pearson’s hard and soft acid−base (HSAB) theory. The soft acid−soft base interaction occurs between Mo^4+^ ions (incomplete bonds on the MoS_2_ QD surface) and the thiol (−SH) group of MPA, forming a Mo^4+^−S­(CH_2_)_2_COO^−^ complex. Conversely, the carboxylate (−COO^−^) groups, which extend outward from the MoS_2_ QDs surface, act as a hard Pearson base and interact with Ti^4+^ ions (incomplete bonds on the TiO_2_ surface), facilitating efficient adsorption and leading to the formation of carboxylate-Ti linkages.^42^ Furthermore, the length of the hydrocarbon chain in MPA plays a crucial role in modulating charge transfer efficiency at the heterojunction interface, influencing the overall electronic properties of the system.^43^

Interface between TiO_2_−MoS_2_ and its chemical composition and surface element valence was further analyzed by X-ray photoelectron spectroscopy (XPS) (Figure 3).

**Figure 3:**
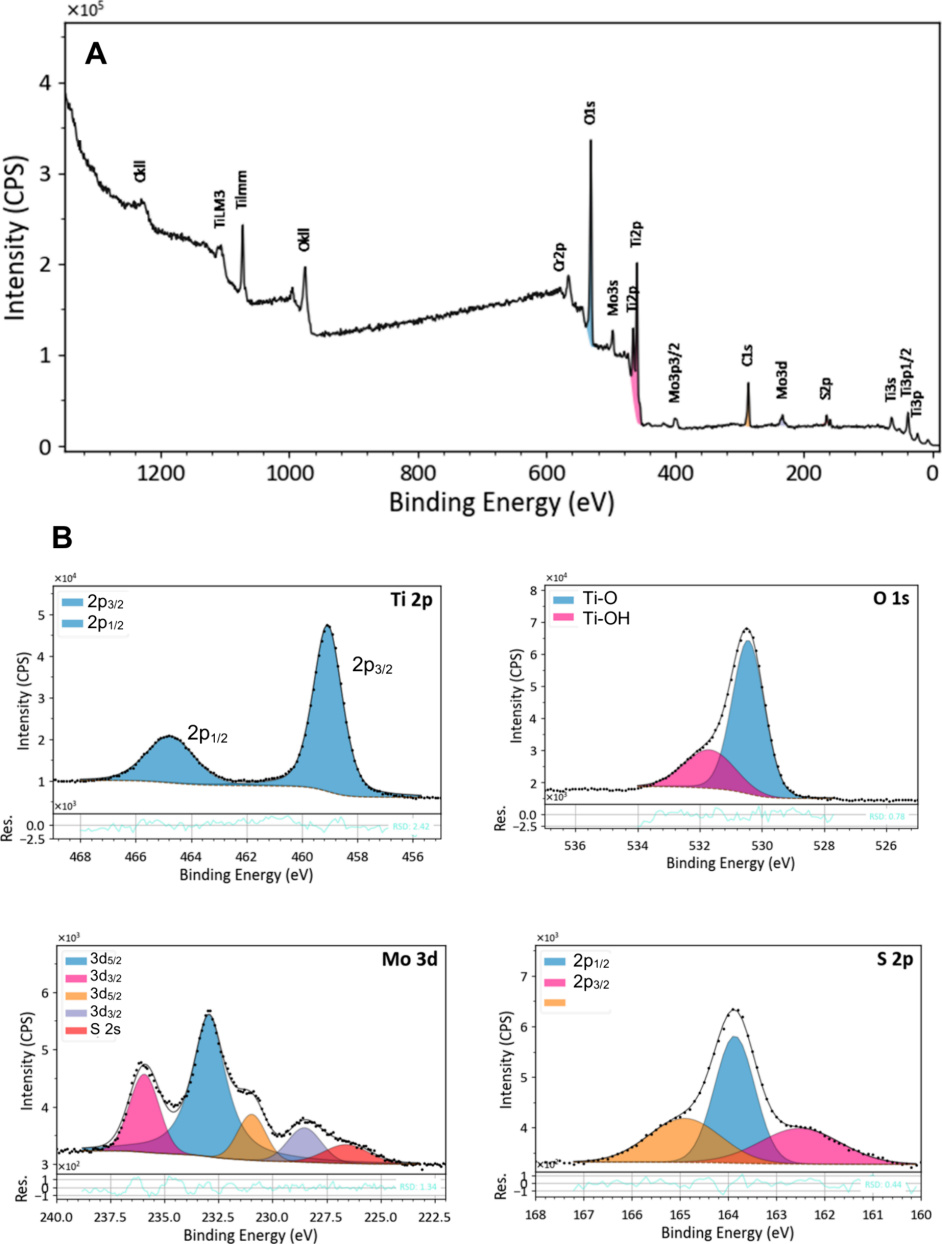
XPS spectra of the TiO_2_ NTs/MoS_2_ QDs interface: (A) survey spectrum and (B) high-resolution XPS spectra of Ti 2p, O 1s, Mo 3d, and S 2p.

The survey spectrum (Figure 3A) confirms the presence of the 1D (TiO_2_ NTs) support and MoS_2_ QDs cocatalysts. Stoichiometric analysis via XPS yielded a Mo:S ratio of 1:2, validating the nanomaterial’s composition. Figure 3B details the high-resolution Ti 2p, O 1s, Mo 3d, and S 2p spectra for the TiO_2_ NTs and MoS_2_ QDs interface. The Ti 2p spectrum exhibits peaks at 459.07 (2p_3/2_) and 464.76 eV (2p_1/2_), confirming the Ti^4+^ state characteristic of the TiO_2_ structure. The O 1s spectrum reveals distinct chemical environments assigned to Ti−O (530.44 eV) and Ti−OH (531.69 eV) bonds. For the Mo 3d spectrum, peaks at 232.94 and 228.52 eV can be indexed to Mo 3d_3/2_ and Mo 3d_5/2_ doublet originated from Mo^4+^, as expected for the MoS_2_ composition. Additional signals observed at 230.96 (3d_5/2_) and 235.93 eV (3d_3/2_) indicated the presence of Mo^6+^ species, which can be associated with the surface oxidation forming MoO_3_ or the interaction of Ti−O−Mo.^19,44^ The S 2p spectrum displays peaks at 163.86 eV (2p_1/2_) and 162.51 eV (2p_3/2_). Thus, these data confirm the decoration of TiO_2_ nanotubes with quantum dots and elucidate the electronic coupling between the semiconductors.

The impact of immersion time on MoS_2_ deposition was initially evaluated by using scanning electron microscopy (SEM) imaging and elemental analysis. Figure 4 shows that relative to the pristine TiO_2_ (Figure 4A), a progressive accumulation of the material on the TiO_2_ nanotube surface occurs with increasing immersion time from 1 h (Figure 4B) to 24 h (Figure 4E). This increased deposition is expected to significantly affect charge transfer dynamics within the heterojunction, potentially enhancing interfacial interactions and improving the overall electronic performance. The presence of MoS_2_ QDs on the TiO_2_ NTs surface was confirmed through energy-dispersive X-ray spectroscopy (EDS) spectra, as shown in Figure 4F. The spectrum presented the S K_α1_ line (2.308 keV) and the Mo L_α1_ (2.293 keV), K_α1_ (17.479 keV), and K_β1_ (19.602 keV) lines, in addition to the signals corresponding to Ti L_α1_ (0.452 keV), K_α1_ (4.515 keV), K_β1_ (4.932 keV), and O K_α1_ (0.525 keV). Despite the overlap of the S K_α1_ and Mo L_α1_ lines, additional Mo-specific peaks were detected, along with a broad baseline in the 1−4 keV region, attributed to other S and Mo transitions. Figure S4 presents the elemental mapping by EDX of TiO_2_ nanotubes coated with MoS_2_ after 24 h of sensitization. The analysis confirms the uniform distribution of MoS_2_ across the TiO_2_ surface, indicating effective adsorption. These observations provide clear evidence of MoS_2_ QDs deposition on the TiO_2_ NTs surface.

**Figure 4:**
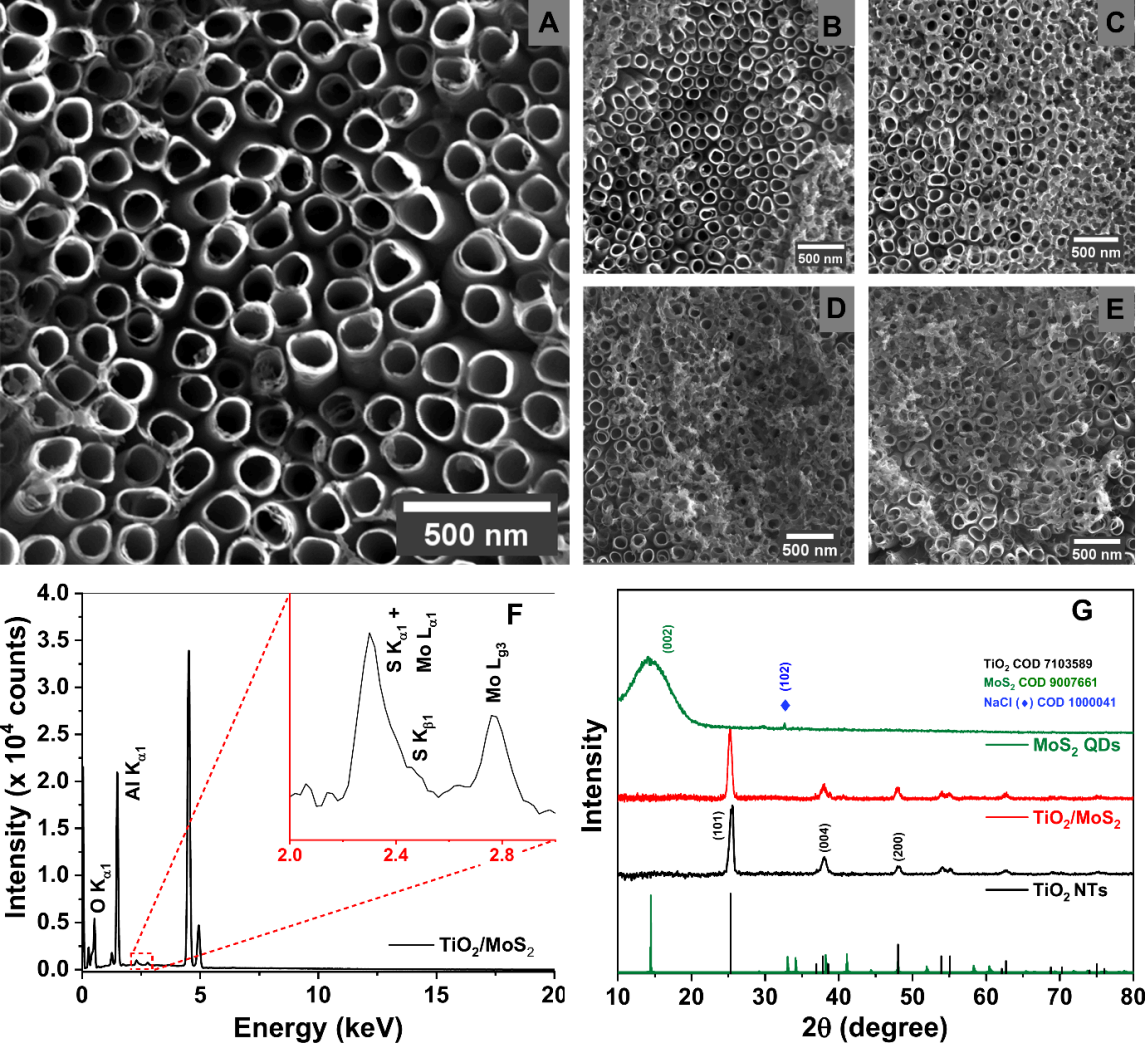
(A) SEM images of TiO_2_ NTs and MoS_2_ QDs-sensitized TiO_2_ NTs samples during different immersion times: (A) pristine, (B) 1 h, (C) 6 h, (D) 12 h, and (E) 24 h. (F) EDX spectrum for TiO_2_/MoS_2_ heterojunction (24 h, sensitization). (G) XRD patterns of TiO_2_ NTs, MoS_2_ QDs, and TiO_2_/MoS_2_ heterojunctions (24 h, sensitization).

XRD patterns (Figure 4G) were obtained to monitor structural modifications at each stage of hierarchical structuring. After anodization, XRD peaks were observed at 2θ values of 25.1°, 37.0°, and 48.0°, corresponding to the (101), (004), and (200) crystallographic planes, respectively. These peaks are indexed to the tetragonal anatase phase of TiO_2_ (COD number 7103589). The low concentration of particles, as observed by the intensity of the peaks in EDX (Figure 4F), does not allow visualization of the system in diffraction, as also observed in previous works.^7^ The interface was characterized by reflectance (Figure S5), which shows an increase in the absorption region into the visible range (Figure S5a) with the deposition of MoS_2_. The TiO_2_ NTs exhibited a bandgap of 3.18 eV, while the TiO_2_/MoS_2_ heterojunction showed a bandgap of 2.97 eV, highlighting the impact of the MoS_2_ QDs in the TiO_2_/MoS_2_ interface.

### 3.3 Electrochemical Characterization and Hydrogen Production

The catalytic activity of the materials toward the hydrogen evolution reaction (HER) was evaluated by using linear sweep voltammetry (LSV) (Figure 5A). A notable shift of 280 mV in the onset potential was observed when comparing pristine TiO_2_ NTs to the TiO_2_/MoS_2_ obtained after 24 h of deposition. However, the voltametric curve reveals reduction processes associated with both the TiO_2_ nanotubes and the MoS_2_ QDs within the same potential range, with peaks centered at approximately 0.75 and 0.45 V, respectively. This overlapping behavior complicates a direct performance comparison between TiO_2_ NTs and the TiO_2_/MoS_2_ heterojunction. To circumvent this issue, a detailed HER performance assessment was conducted at a higher overpotential (η^100^). The optimized TiO_2_/MoS_2_ (24h) electrode exhibited a η^100^ value of 617 mV, with other deposition conditions showing values approximately 30 mV higher. In contrast, the pristine TiO_2_ NTs showed a significantly higher η^100^ value of 927 mV, underscoring their poor electrocatalytic activity.

These findings are further corroborated by electrochemical impedance spectroscopy (EIS) data (Figure 5B).^45^ The EIS data demonstrate a substantial decrease in charge transfer resistance *R*_ct_ from 475 Ω (TiO_2_ NTs) to 9.9 Ω (TiO_2_/MoS_2_ 24 h), indicating enhanced charge transfer kinetics upon MoS_2_ deposition. The decrease in the electron transfer resistance, observed in the Nyquist plot, can be correlated with the interfaces verified in the SEM images (Figure 4A−E). With the increase in the amount of MoS_2_ deposited onto the surface of the TiO_2_ NTs, a reduction in the electron transfer resistance at the interface with the electrolyte solution was observed. This suggests that the hierarchical structuring of the nano- and microstructures enhances the electrocatalytic performance.

Tafel slope analysis (Figure 5C) revealed a slope value of 106 mV dec^−1^ for the TiO_2_ NTs/MoS_2_ QDs (24 h) sample, suggesting that the Volmer step (electrochemical hydrogen adsorption) is the rate-determining step in an alkaline medium.^46,47^

**Figure 5:**
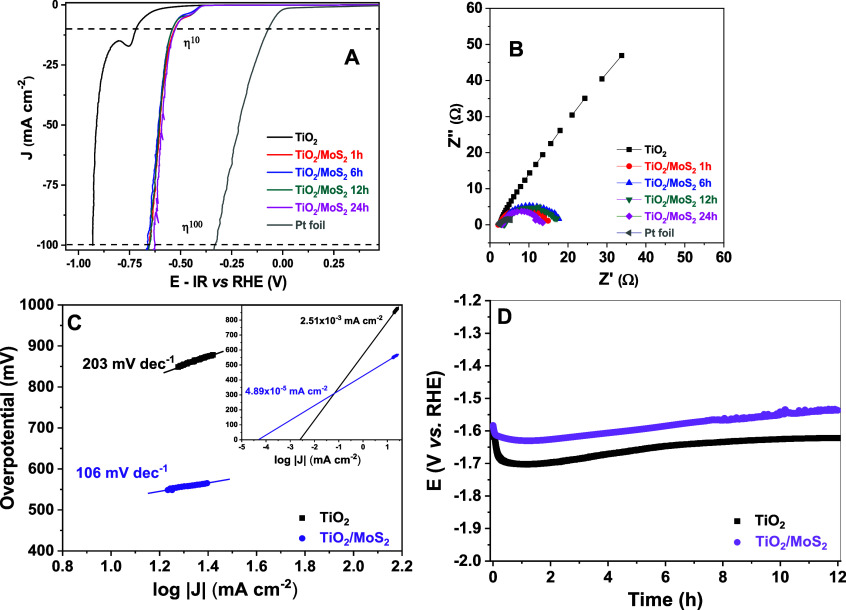
(A) Linear sweep voltammograms comparing the pristine TiO_2_ NTs electrode, TiO_2_ NTs/MoS_2_ QDs composite electrodes at different deposition times (1, 6, 12, and 24 h), and the Pt foil for HER in KOH 1.0 mol L^−1^ KOH. 100% IR compensation was applied to eliminate ohmic losses. (B) Nyquist plot from the EIS measurements comparing the TiO_2_ NTs electrode and TiO_2_ NTs/MoS_2_ QDs electrodes at different immersion times (1, 6, 12, and 24 h) at the onset potential of −0.477 V vs RHE and the Pt foil at −0.1 V RHE in KOH 1.0 mol L^−1^. (C) Tafel slope comparison between the pristine TiO_2_ NTs electrode and the TiO_2_ NTs/MoS_2_ QDs (24 h) electrodes, highlighting the improvement in catalytic kinetics. Inset: Extrapolation of the Tafel curves determines the exchange current density (J_0_) parameter, providing insights into the intrinsic electrocatalytic activity. (D) Stability test of TiO_2_ NTs and TiO_2_/MoS_2_ electrode: Time-dependent cathodic chronopotentiometry was performed during the HER process over 12 h at 10 mA cm^−2^ (after *iR* compensation).

In contrast, the pristine TiO_2_ NTs electrode exhibited a high Tafel slope of 203 mV dec^−1^, indicating sluggish HER kinetics due to its limited charge transfer efficiency. This analysis highlights the enhanced catalytic activity imparted by MoS_2_ QDs deposition, as evidenced by the significant reduction of *R*_ct_ and the more favorable Tafel slope. These improvements collectively indicate a substantial improvement in HER performance compared to the bare TiO_2_ NTs electrode.^48^ The stability of an electrocatalyst is a critical parameter for its potential commercialization as it determines its long-term activity retention under operational conditions. Therefore, evaluating the stability of TiO_2_/MoS_2_ during the hydrogen evolution reaction (HER) is essential.^49–52^ The stability test was conducted using a chronopotentiometric study over 12 h at a constant current density of −10 mA cm^−2^, as shown in Figure 5D. The results demonstrate that both electrodes exhibit reasonably good stability, with a slight performance decline observed for the pristine TiO_2_ NTs electrode. Notably, the TiO_2_ electrode required a higher overpotential compared to the TiO_2_/MoS_2_ heterojunction, confirming that MoS_2_ QD deposition significantly enhances surface coverage and improves HER efficiency by facilitating charge transfer and catalytic activity. Platinum-based electrolyzers have been commercially viable since 1888, with the first large-scale electrolyzer achieving a capacity of 10,000 m^3^ H_2_ h^−1^ in 1939.^53^ While advancements have been made in pressurization systems and membrane technologies, a major challenge remains: the development of cost-effective electroactive materials derived from earth-abundant elements and powered by renewable energy sources.

The H_2_ evolution results are presented in Figure 6. The performance demonstrates a significant improvement in hydrogen production, where TiO_2_ produced 255.0 μmol cm^−2^ h^−1^ (6.0 mL cm^−2^ h^−1^) H_2_, while TiO_2_/MoS_2_ achieved 602.5 μmol cm^−2^ h^−1^ (13.5 mL cm^−2^ h^−1^). These results confirm a 2.3-fold increase in hydrogen production for TiO_2_/MoS_2_ compared to TiO_2_, further validating the photoelectrochemical enhancements introduced by MoS_2_ QDs deposition.

**Figure 6:**
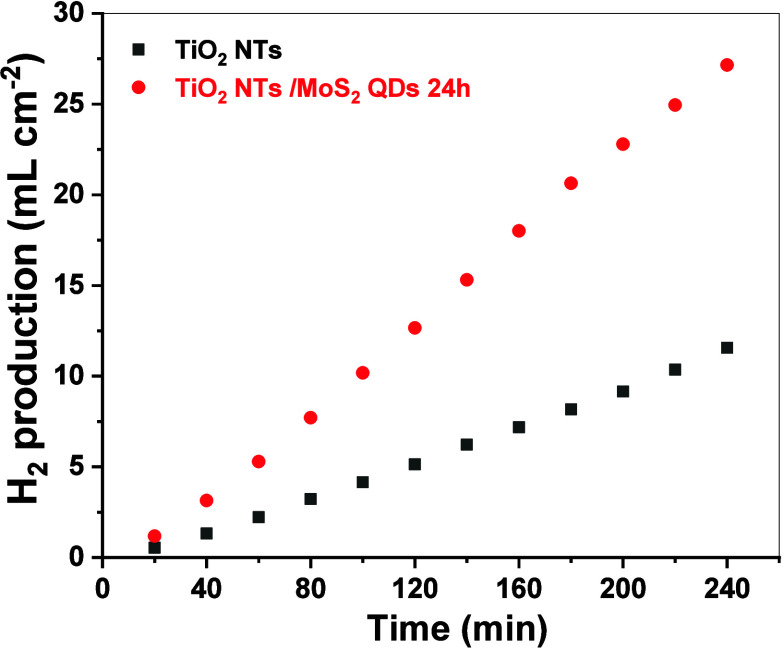
Profile HER evolution to TiO_2_ NTs and TiO_2_ NTs/MoS_2_ QDs electrodes.

One of the merits of this work is the reporting of the hydrogen production rate in an alkaline medium using KOH as the electrolyte. Since this is a well-established technology, the development of electrodes needs to compare their performance with commercially available electrodes, most of which are based on platinum. A comparison of the performance of the developed electrodes relative to other systems reported in the literature is presented in Table S1. The TiO_2_/MoS_2_ heterojunction for electrocatalytic hydrogen generation has been proposed through various strategies, including the preparation of TiO_2_ nanotubes supported by anodization and decorated with MoS_2_ via hydrothermal methods,^5,39^ cathodic electrodeposition,^54^ MOF-template,^55^ particle electrodeposition,^56^ and photoinduced deposition.^57^ TiO_2_/MoS_2_ systems deposited on glassy carbon have also been reported, where an inverse architecture with MoS_2_ nanotubes decorated with TiO_2_ was also evaluated.^58^ Commonly, studies have reported the use of sulfuric acid as electrolytes, which allows for comparison; however, such aggressive acidic conditions can damage the electrocatalysts and reduce their lifetime. The literature reports average overpotentials (η^10^) of 174 mV vs RHE using 0.5 mol L^−1^ H_2_SO_4_. In contrast, in the present work, we report an overpotential (η^100^) of 617 mV vs RHE, which is a highly significant result.

The Tafel slope (106 mV dec^−1^) and overpotential required to reach a cathodic current density of 100 mA cm^−2^ in this work exhibit values comparable to those reported for seven other catalyst systems based on TiO_2_/MoS_2_ (η^10^) that used acidic conditions. The overpotential (η^100^@ - 617 mV vs RHE) and Tafel slope (106 mV dec^−1^) for the Ti/TiO_2_/MoS_2_ system demonstrate the significant electrocatalytic activity of the system developed via the heterojunction of nanostructures by immersion.

Some works reporting heterojunctions between MoS_2_ and various nanomaterials for electrocatalytic hydrogen generation (Table S2) include MoS_2_ ultrathin nanosheets interlaced on carbon spheres, which achieved an η^10^ of 106 mV and a Tafel slope of 53 mV dec^−1^.^59^ Additionally, M-NiS/Mo_2_S_3_ (M = Co, Fe, Ce, and Bi) demonstrated an η^10^ of 142 mV when operating in an alkaline medium (1.0 mol L^−1^ KOH).^60^ These results are consistent with the findings presented in the current work.

### 3.4 Kinetic Modeling and Experimental Validation

Given the well-established models for hydrogen evolution kinetics on electrode surfaces (Volmer, Heyrovsky, and Tafel mechanisms),^61^ a validation process was adopted to reconcile the indicators obtained from experimental results for hydrogen evolution on the TiO_2_/MoS_2_ surface with the classical kinetic equations.^62^ A program was developed in Python using the Pycharm software (version: 2024.3.1, build: 243.22562.180) for kinetic modeling to adjust the results to different classical kinetic equations based on the classical zero-order, first-order, and second-order kinetic equations (eq 7):

$$\frac{d[A]}{dt}=k[A{]}^{2}\;∴\;\frac{1}{\left[A\right]}=\frac{1}{[A{]}_{0}}+kt$$ 7

where [*A*]_(*t*)_ is the concentration of the species over time, [A]_0_ is the initial concentration, *k* is the rate constant, and t is the time. The results do not match between the experimental data and zero-order and first-order equations due to *R* ^2^ = 0.534 for zero-order and *R* ^2^ = 0.609 for first-order. The second-order model was identified as the one that best described the HER (*R* ^2^ = 0.994), even when the relationship between the inverse of the concentration and time was not linear (Figure 7).

**Figure 7:**
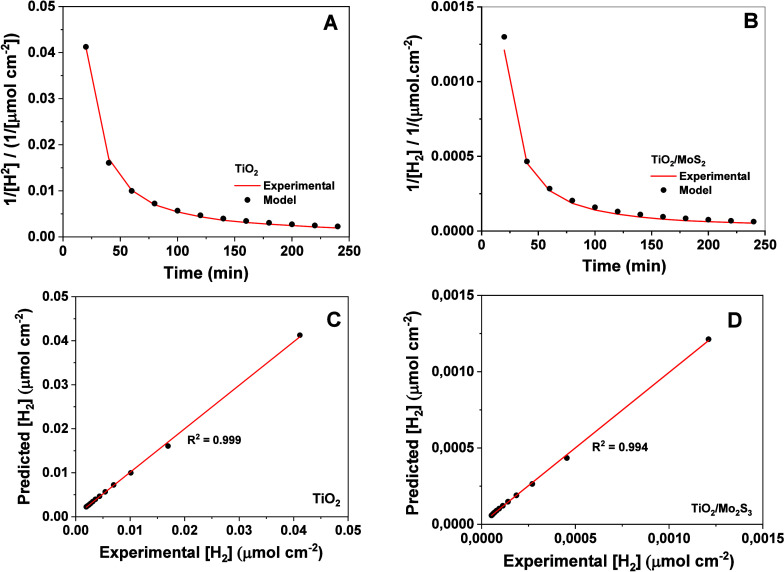
Kinetic modeling of hydrogen production for (A) TiO_2_ and (B) TiO_2_/MoS_2_ electrodes. Model validations for (C) TiO_2_ and (D) TiO_2_/MoS_2_ electrodes.

This nonlinearity was investigated and explained based on complex factors influencing HER kinetics in electrocatalytic systems. The Volmer, Heyrovsky, and Tafel mechanisms consider different ways for charge transference, adsorption, and reactional products since the mechanisms can be verified simultaneously.^63^ The presence of multiple adsorbed intermediates and the competition for active sites on the electrode surface turn the reaction mechanism complex.^64,65^ Catalytic surface changes, such as corrosion and dissolution, can occur during continuous operation of the electrocatalyst, leading to alterations in catalytic properties and a possible transition in the reaction mechanism, highlighting that these changes can result in a nonlinear relationship between the inverse of concentration and time, influencing the observed.

The graphs generated by the second-order kinetic model for the TiO_2_ and TiO_2_/MoS_2_ electrodes are represented in Figure 7A,B, respectively. In systems where the surface is highly covered by intermediates or where multiple steps determine the kinetics, Tafel slopes and reaction orders may not reflect the expected linear relationship for second-order reactions.^64^ This profile supported this conclusion, showing excellent agreement with the experimental data. Furthermore, the analysis of the correlation between the experimental results and the values calculated by the model showed a coefficient of determination (*R* ^2^) around 0.999 for TiO_2_ (Figure 7C) and 0.994 for TiO_2_/MoS_2_ (Figure 7D). This high value confirms the accuracy of the second-order model in describing HER kinetics in the TiO_2_/MoS_2_ system, reinforcing the interpretation of a “pseudo-second-order” kinetics, where surface processes or mass transport play a crucial role in the reaction rate due to the complex adsorption of chemical species onto the electrocatalyst surface.

## 4 Conclusions

This study successfully demonstrates the synthesis and characterization of a TiO_2_ NTs/MoS_2_ QDs heterojunction, resulting in a 2.3-fold enhancement in HER activity compared to bare TiO_2_ (12 mL of H_2_ cm^−2^ for TiO_2_ NTs). Structural analyses by XRD and TEM confirm the formation of a well-defined heterojunction with MoS_2_ QDs uniformly distributed across the TiO_2_ NTs surface. Electrochemical analyses further validate the improved HER kinetics and reduced charge transfer resistance facilitated by heterojunction formation. Tafel slope analysis highlights the enhanced catalytic efficiency, where the pristine TiO_2_ NTs exhibited an η^100^ value of 927 mV, while the TiO_2_ NTs/MoS_2_ QDs heterojunction achieved an η^100^ of 617 mV. These findings indicate that the heterojunction favors the Volmer mechanism as the rate-determining step under alkaline conditions. Kinetic modeling suggests a pseudo-second-order mechanism, influenced by surface processes, and multiple adsorbed intermediates, contributing to the observed electrocatalytic performance. Characterization techniques, including XRD, TEM, UV−vis, and Raman spectroscopy, further support the efficient charge separation and heterojunction formation, crucial for enhancing catalytic activity. This work highlights the promising potential of TiO_2_ NTs/MoS_2_ QDs heterojunctions as efficient and scalable electrocatalysts for sustainable hydrogen production. Future research efforts should focus on optimizing deposition strategies and exploring other transition metal dichalcogenide nanostructures to further advance renewable energy applications.

## Supplementary Material



## Notes

Absorption spectra and Tauc plot, SEM images and EDX spectra, and comparative table between the data reported in the literature and those presented in the work (PDF)
