# Supplementary material for: Boosting Hydrogen Evolution Kinetics with MoS2-Decorated TiO2 Nanotubes
Source: ACS Omega. 2026 Mar 25;11(13):20079–90. doi: 10.1021/acsomega.5c06367 (PMC13063172; doi:10.1021/acsomega.5c06367)
Supplement: Supplementary file 1 [file ao5c06367_si_001.pdf]

Supporting information

Boosting Hydrogen Evolution Kinetics with MoS_2-Decorated TiO_2 Nanotubes

Leonardo J. L. Maciel,[1] Denilson V. Freitas,[1] Felipe L. N. Sousa,[1] Luana B. C. Oliveira,[1,2] Otávio A. L. Alves[1,2], Francisco de A.S. Ribeiro,[1,2] Giovanna Machado[1*]

[1] Centro de Tecnologias Estratégicas do Nordeste (CETENE), 50740-540 Recife, PE, Brazil.

[2] Universidade Federal de Pernambuco (UFPE), 50670-901, Recife, PE, Brazil.

* Corresponding author: giovanna.machado@cetene.gov.br (G. Machado)

* Brazilian Chemical Society member

Summary

	Pag.
Figure S1: The electrochemical cavity cell	S3
Figure S2: (a) UV-Vis spectrum of MoS_2 QDs for the sample after dialysis and different heating times (b) Band Gap energy for TiO_2 and TiO_2@Mo_2S_3 QDs (c) Evolution of the Band Gap energy over time for the Mo_2S_3 QDs sample after different heating times.	S4
Figure S3: FTIR spectrum for TiO_2 nanotubes and TiO_2 NTs/ MoS_2 QDs	S5
Figure S4: (a) Absorption spectra acquired by reflectance and (b) Tauc plot extrapolation.	S5
Figure S5: (a) SEM cross-section micrograph and (b) EDX elemental mapping of TiO_2/MoS_2 heterojunction.	S6
Table S1: Comparison of electrochemical performance of TiO_2/MoS_2 systems reported in the literature.	S7
Table S2: Comparison of electrochemical performance of heterojunctions with Mo_2S_3 reported in the literature.	S8
References	S9

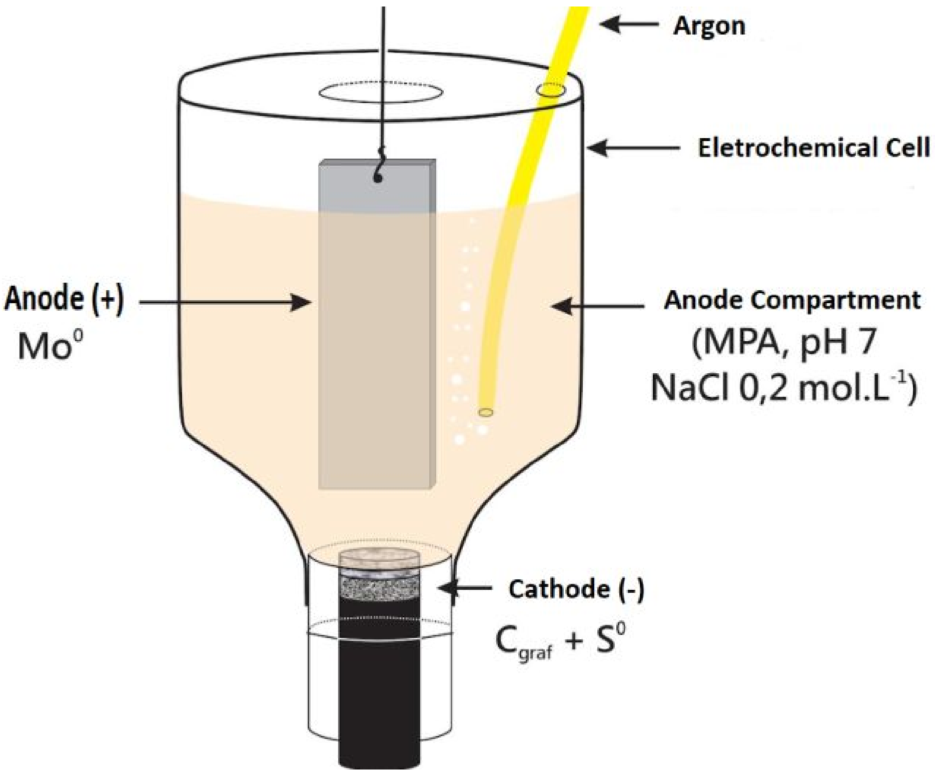

Figure S1: The electrochemical cavity cell

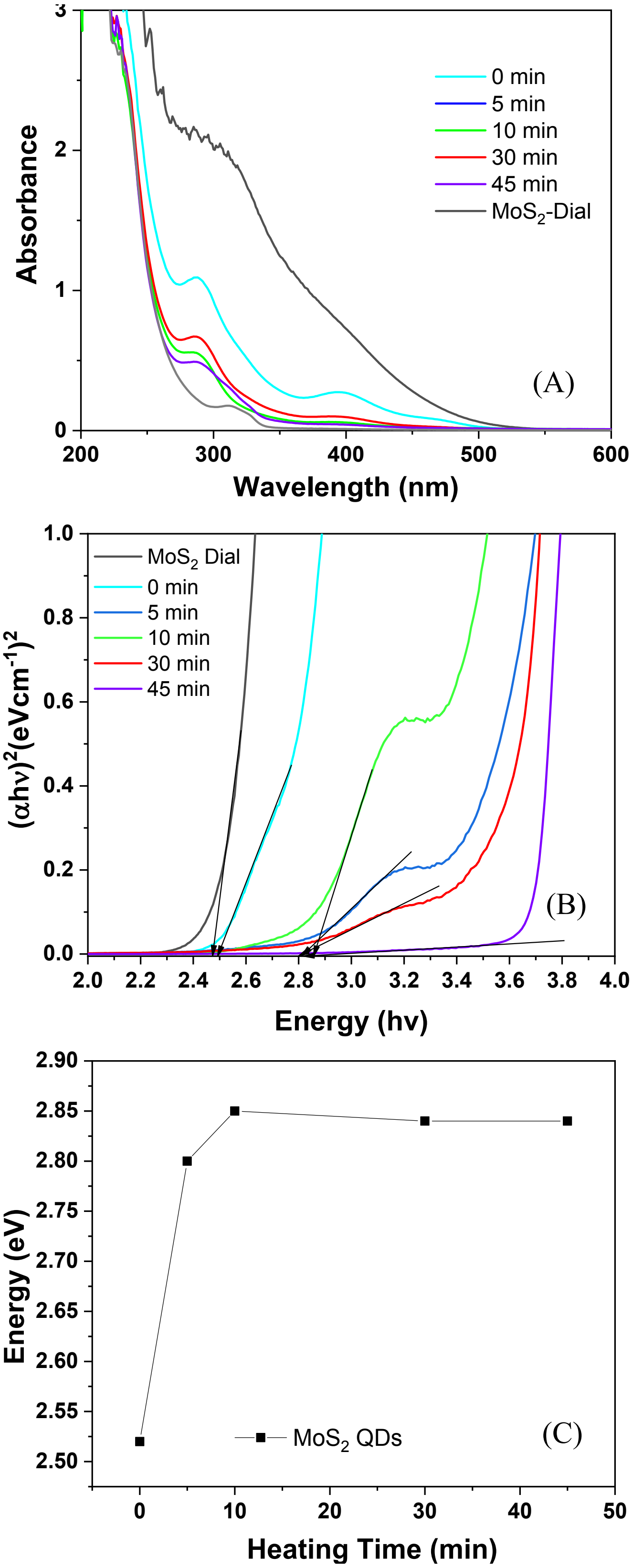

Figure S2: (A) UV-Vis spectrum of MoS_2 QDs for the sample after dialysis and different heating times (B) Band Gap energy for TiO_2 and TiO_2 NTs / MoS_2 QDs (C) Evolution of the Band Gap energy over time for the MoS_2 QDs sample after different heating times.

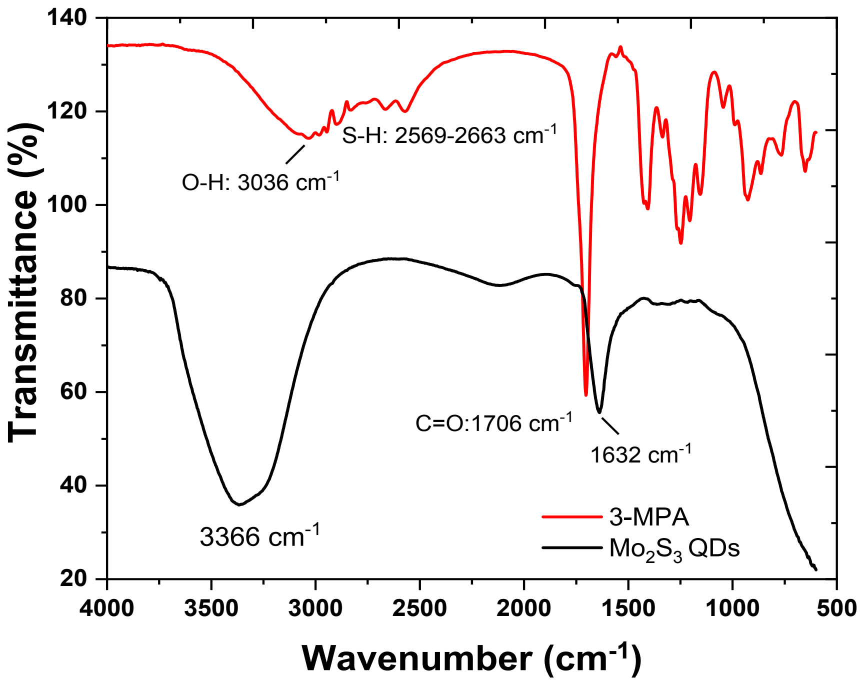

Figure S3: FTIR spectrum for TiO_2 nanotubes and TiO_2 NTs/ MoS_2 QDs.

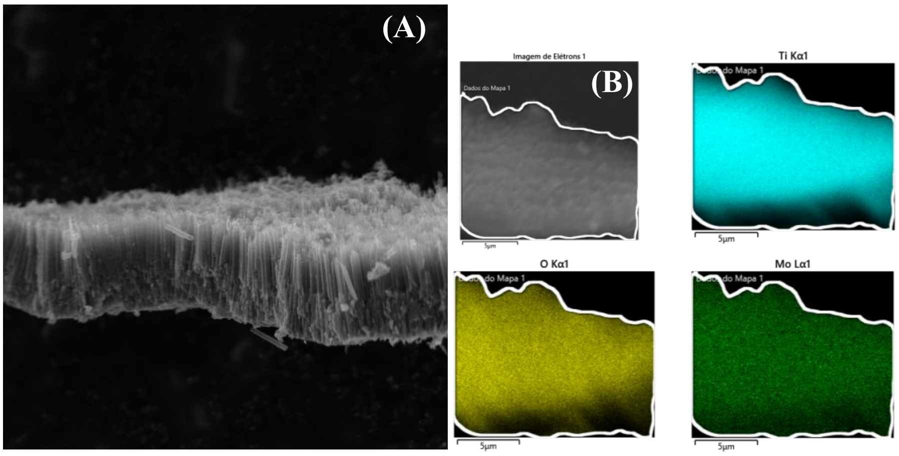

Figure S4: (a) SEM cross-section micrograph and (b) EDX elemental mapping of TiO_2/ MoS_2 heterojunction.

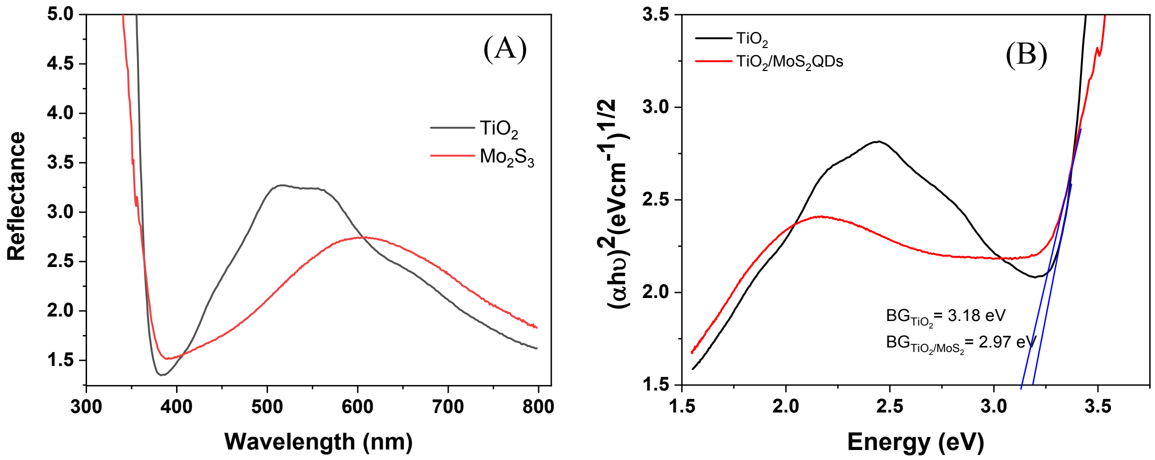

Figure S5: (A) Absorption spectra acquired by reflectance and (B) Tauc plot extrapolation.

Table S1: Comparison of electrochemical performance of TiO_2/MoS_2 systems reported in the literature.

**Reference**	**Materials**	**Method**	**Electrolyte**	**H_2 evolution rate (mL cm^{-2} h^{-1})**	**η^{10} (mV vs. RHE)**	**Tafel slope** (mV dec^{-1}**)**
[1]	Ti foil / TiO_2 NTs / MoS_2	Electrosynthesis and *in situ* growth of MoS_2 by hydrothermal.	0.5 mol L^{-1} H_2SO_4	-	200	50
[2]	Ti foil / TiO_{2-x} NTs / MoS_2	Electrosynthesis and thermal activation, and *in situ* growth of MoS_2 by hydrothermal.	0.5 mol L^{-1} H_2SO_4	-	146	42
[3]	MoS_2 NTs / TiO_2 NPs supported in glass carbon	Solvothermal and *in situ* growth of TiO_2 by hydrothermal.	0.5 mol L^{-1} H_2SO_4	-	210	42
[4]	Ti foil / TiO_2 NTs / MoS_2	Electrosynthesis and electrodeposition of MoS_2 by cathodic procedure.	0.5 mol L^{-1} H_2SO_4	-	93	43
[5]	MoS_2-TiO_2 NPs supported in glass carbon	MOF- NH_2MIL125(Ti) template by hydrothermal followed by the *in situ* growth of MoS_2 by hydrothermal.	0.5 mol L^{-1} H_2SO_4	-	300	81
[6]	Ti foil / TiO_2 NTs / MoS_2	Electrosynthesis and photoinduced MoS_2 deposition.	0.5 mol L^{-1} H_2SO_4	-	114	52
[7]	Amorphous MoS_{x-2} Coated TiO_2	Electrosynthesis and electrodeposition of MoS_2.	0.5 mol L^{-1} H_2SO_4	-	157	53
This work	Ti foil / TiO_2 NTs / MoS_2 QDs	Electrosynthesis and electrochemical production of MoS_2, and adsorption by immersion.	1.0 mol L^{-1} KOH	13.5	η^{100}@ - 617	106

Table S2: Comparison of electrochemical performance of heterojunctions with Mo_2S_3 reported in the literature.

**Reference**	**Materials**	**Method**	**Electrolyte**	**H_2 evolution rate (mL cm^{-2} h^{-1})**	**η^{10} (mV vs. RHE)**	**Tafel slope (mV dec^{-1})**
[8]	Mo_2S_3 ultrathin nanosheets interlacedly on carbon spheres	Hydrothermal synthesis.	-	-	106	53
[9]	M-NiS/Mo_2S_3 (M = Co, Fe, Ce and Bi)	Hydrothermal synthesis	1.0 mol L^{-1} KOH	-	142	-
This work	Ti foil / TiO_2 NTs / MoS_2 QDs	Electrosynthesis and electrochemical production of MoS_2, and adsorption by immersion.	1.0 mol L^{-1} KOH	13.5	η^{100}@ - 617	106

References

[1] AlAqad, K. M.; Kandiel, T. A.; Basheer, C. Synergy between In-Situ Immobilized MoS_2 Nanosheets and TiO_2 Nanotubes for Efficient Electrocatalytic Hydrogen Evolution. *Int. J. Hydrogen Energy* **2022**, *47* (4), 2366–2377. https://doi.org/10.1016/j.ijhydene.2021.10.159.

[2] Zhang, T.; Yang, T.; Qu, G.; Huang, S.; Cao, P.; Gao, W. Phase Control and Stabilization of 1T-MoS_2 via Black TiO_{2-x} Nanotube Arrays Supporting for Electrocatalytic Hydrogen Evolution. *J. Energy Chem.* **2022**, *68*, 71–77. https://doi.org/10.1016/j.jechem.2021.10.031.

[3] Feng, B.; Liu, C.; Yan, W.; Geng, J.; Wang, G. MoS_2 Nanotubes Loaded with TiO_2 Nanoparticles for Enhanced Electrocatalytic Hydrogen Evolution. *RSC Adv*. **2019**, *9* (45), 26487–26494. https://doi.org/10.1039/C9RA05041H.

[4] Medina, M.; Corradini, P. G.; de Brito, J. F.; Sousa Santos, H. L.; Mascaro, L. H. The Substrate Morphology Effect for Sulfur-Rich Amorphous Molybdenum Sulfide for Electrochemical Hydrogen Evolution Reaction. *J. Electrochem. Soc.* **2022**, *169* (2), 026519. https://doi.org/10.1149/1945-7111/ac5067.

[5] Ma, B.; Guan, P.-Y.; Li, Q.-Y.; Zhang, M.; Zang, S.-Q. MOF-Derived Flower-like MoS_2 @TiO_2 Nanohybrids with Enhanced Activity for Hydrogen Evolution. *ACS Appl. Mater. Inter.* **2016**, *8* (40), 26794–26800. https://doi.org/10.1021/acsami.6b08740.

[6] Meng, C.; Liu, Z.; Zhang, T.; Zhai, J. Layered MoS_2 Nanoparticles on TiO_2 Nanotubes by a Photocatalytic Strategy for Use as High-Performance Electrocatalysts in Hydrogen Evolution Reactions. *Green Chem.* **2015**, *17* (5), 2764–2768. https://doi.org/10.1039/C5GC00272A.

[7] Liu, Z.; Zhang, X.; Wang, B.; Xia, M.; Gao, S.; Liu, X.; Zavabeti, A.; Ou, J. Z.; Kalantar-Zadeh, K.; Wang, Y. Amorphous MoS_x -Coated TiO_2 Nanotube Arrays for Enhanced Electrocatalytic Hydrogen Evolution Reaction. *J. Phys. Chem. C* **2018**, *122* (24), 12589–12597. https://doi.org/10.1021/acs.jpcc.8b01678

[8] Lili Bo, Lumei Pu, Yusen Hu, Fang Nian, Zhixia Zhang, Ping Li, Jinhui Tong. Hydrangea like composite catalysts of ultrathin Mo2S3 nanosheets assembled on N, S-dual-doped graphitic biocarbon spheres with highly electrocatalytic activity for HER. Int. J. Hydrogen Energy 2022, 47 (10), 6700-6709. https://doi.org/10.1016/j.ijhydene.2021.12.042.

[9] Han Zhao, Min Liu, Xiaoqiang Du, Xiaoshuang Zhang. Synthesis of M-NiS/Mo2S3 (M=Co, Fe, Ce and Bi) nanoarrays as efficient electrocatalytic hydrogen evolution reaction catalyst in fresh and seawater. Int. J. Hydrogen Energy 2024, 62, 532-540. https://doi.org/10.1016/j.ijhydene.2024.03.077.
